# The Treatment of Tuberculosis with Urea

**Published:** 1901-10

**Authors:** 


### The Treatment of Tuberculosis with Urea.

Arthur A. Buch, F. R. C. S., in the *Philadelphia Medical Journal,* discusses the urea treatment of tuberculosis as suggested by Dr. Harper, of Nottingham.

He says that most individuals possess some vito-chemical combination in their systems which renders them immune at any ordinary dose of infection by the tubercle bacillus, hence in spite of frequent exposures comparatively few contract tuberculosis. Whatever this substance or combination is, it must be such that it is neither harmful nor foreign to the human organism.

It must exist to a greater degree in some than in others, and if we could induce changes in the susceptible by which this substance could be produced in greater amount we could thereby rid the world of tuberculosis. Dr. Harper observing gouty persons to be peculiarly immune to tuberculosis began the experiment of administering urea to tuberculous patients, and with wonderfully successful results. It is interesting to note that in the laboratory the tubercle bacillus grows best on media of low nitrogenous value, and that among the lower animals, the herbiverous are more susceptible to tuberculosis than are the carniverous. The cow well illustrates the susceptibility of herbiverous animals.

